# Unleashing the Potential for Patient-Generated Health Data (PGHD)

**DOI:** 10.1007/s11606-023-08461-4

**Published:** 2024-01-22

**Authors:** Kim M. Nazi, Terry Newton, Christina M. Armstrong

**Affiliations:** 1 Trilogy Federal, LLC, Arlington, VA USA; 2 KMN Consulting Services, LTD, Coxsackie, NY USA; 3 Trilogy Federal, LLC, 44 Mountain View Drive, Coxsackie, NY 12051 USA; 4 Office of Connected Care, US Department of Veterans Affairs, Washington, DC USA

## Abstract

Patient-generated health data (PGHD) is data created, captured, or recorded by patients in between healthcare appointments, and is an important supplement to data generated during periodic clinical encounters. PGHD has potential to improve diagnosis and management of chronic conditions, improve health outcomes, and facilitate more “connected health” between patients and their care teams. Electronic PGHD is rapidly accelerating due to the proliferation of consumer health technologies, remote patient monitoring systems, and personal health platforms. Despite this tremendous growth in PGHD and anticipated benefits, broadscale use of PGHD has been challenging to implement with significant gaps in current knowledge about how PGHD can best be employed in the service of high-quality, patient-centered care. While the role of PGHD in patient self-management continues to grow organically, we need a deeper understanding of how data collection and sharing translate into actionable information that supports shared decision-making and informs clinical care in real-world settings. This, in turn, will foster both clinical adoption and patient engagement with PGHD. We propose an agenda for PGHD-related research in the Veterans Health Administration that emphasizes this clinical value to enhance our understanding of its potential and limitations in supporting shared decision-making and informing clinical care.

## INTRODUCTION

Patient-generated health data (PGHD) is data created, captured, or recorded by patients between healthcare appointments and is an important supplement to the data generated during periodic clinical encounters.^1^ PGHD can include data about the patient’s experience, health or treatment history, symptoms, biometric data, vital signs, or ability to function and move that may be tracked by any method (e.g., mobile apps, sensors, devices). PGHD can support the ability to diagnose and manage chronic conditions, and has the potential to improve health outcomes, and to facilitate more “connected health” between patients and their care teams.^2^ This data can add significant depth and dimension to a clinician’s understanding of a patient’s health, which, in turn, can lead to improved clinical decision-making and better care delivery.^3, 4^ It can also benefit patients in understanding their health and treatment options, and in self-management of symptoms.^5, 6^ For example, patients reported that tracking their observations of daily living (ODLs), such as exercise, level of stress, and nutrition patterns, helped them to gauge how they were progressing, guide them in their choices of health actions, and determine if the actions they had taken were producing the desired effect (e.g., noting how daily food choices contribute to fluctuations in blood pressure).^5^

Research suggests that PGHD has the potential to improve patient quality of life,^7^ promote healthy behaviors,^8^ increase patient activation,^9^ and enhance patient empowerment and engagement.^10^ Further engaging patients in their own care, in turn, has the potential to improve the patient experience and increase satisfaction,^11^ enable patients to achieve better health outcomes,^12, 13^ and enhance system efficiency, thereby reducing cost.^14, 15^

While receiving data and information from patients is not new for clinicians, the amount and availability of electronic PGHD are rapidly accelerating due to the proliferation of consumer health technologies, remote patient monitoring systems, and personal health platforms that enable patients to record or contribute data. Despite this tremendous growth in PGHD and anticipated benefits, broadscale use of PGHD has been challenging to implement with significant gaps in current knowledge about how PGHD can best be employed in the service of high-quality, patient-centered care.^16^ PGHD implementation challenges include clinician and patient burden, usability issues, and clinical workflow integration. The American College of Cardiology Roadmap for Innovation task force report concluded that use of PGHD in clinical practice is in the promising stage but needs further work for widespread adoption and seamless integration into healthcare systems.^17^

## VHA CONNECTED CARE AND PGHD

The Veterans Health Administration (VHA) of the Department of Veterans Affairs (VA) is the largest and most geographically diverse integrated healthcare system in the USA, providing healthcare services to more than nine million Veterans.^18^ VHA has been a pioneer in the use of technology to deliver healthcare services and empower Veterans with virtual care tools and resources. This “connected care” approach includes the development of electronic health resources within VHA’s Office of Connected Care (OCC) including a tethered personal health record patient portal (My Health***e***Vet),^19^ mobile and web applications (e.g., Share My Health Data),^20^ an automated text messaging service (Annie),^21^ and a comprehensive telehealth program to provide virtual healthcare and remote patient monitoring,^22^ with more than 20 VHA standardized disease management protocols (e.g., hypertension, diabetes, heart failure, depression).^23^ Many additional VHA virtual care technologies now support the collection and sharing of PGHD.^24^

VHA OCC, in partnership with other VHA stakeholders, has adopted a broad definition of PGHD, based on a minor adaptation of the 2018 definition provided by the Department of Health and Human Services Office of the National Coordinator for Health Information Technology (ONC):^1^

PGHD is health-related data created, recorded, or gathered by or for patients (or family members or other caregivers) to promote health and wellness or to help address a health concern. PGHD may include remote device-generated consumer collected clinical data, health history, treatment history, biometric data, symptoms, and other types of patient-reported outcomes (PROs), perceived quality of life data, and patient goals, values and lifestyle choices. Health-related PGHD are distinct from data generated in clinical settings. Patients are primarily responsible for capturing and/or recording these data, and patients explicitly decide to share these data through some form of authorization or consent process, or by voluntarily sending or transmitting their data.

A 2018 environmental scan of PGHD in the VHA data ecosystem demonstrated a broad array of current and planned PGHD applications, systems, and use cases, including mobile applications,^24^ and remote patient monitoring using biometric sensors such as daily foot temperature readings for diabetic patients.^25^ While competencies for the use of sensors, wearables, and remote patient monitoring in clinical care and training have been established,^26^ the “standard of care” for PGHD usage has not yet been integrated into standard clinical practice. Effective strategies are needed for managing what data is collected, how and where that data is stored, which data is sent to the clinical care team, and professional responsibility for review or action. A comprehensive VHA organizational strategy is needed to realize and sustain anticipated benefits; minimize risks; ensure compliance with regulations, policies, and standards; and enable a consistent and engaging Veteran experience. Strategic priorities include an enterprise-wide VHA PGHD policy coupled with a VHA PGHD Operations Manual to ensure consistent practices in the collection, review, and use of PGHD across VHA settings of care.

From 2018 to 2022, VA OCC conducted a three-phase approach to develop an organizational strategy for enabling patient-generated health data (PGHD) including literature reviews, whitepapers, best practices research, a decision-making framework, and a recommended governance structure. The VHA OCC PGHD Cross Functional Workgroup was created in 2022 to serve as a multidisciplinary resource (e.g., informatics, innovations, clinicians) to surface and address PGHD-related functional, operational, and policy issues; to develop effective processes; and to support innovation. The workgroup has proposed VHA general guidelines for the collection, sharing, and use of PGHD. These guidelines describe important considerations including categorizing different types of PGHD as *unsolicited* (e.g., a patient tracks their health data for their own purposes), *solicited* (e.g., a clinician requests that a patient collect and submit certain data, or prescribes a device or technology as part of an established program or protocol along with documentation of that agreement in a clinical progress note), or *suggested* (e.g., a clinician recommends or suggests that a patient collect and share PGHD with a common agreement about how that data will be reviewed and used along with documentation of that agreement in a clinical progress note). Examples are shown in Table 1.

**Table 1:** Examples of PGHD Categories

Unsolicited PGHD	A patient downloads a mobile application or logs into a web portal and chooses to connect their wearable biometric device or manually enter their health data (e.g., glucose, weight, blood pressure reading, pulse oximetry). The patient may or may not inform their provider of this activity, but the patient wants their healthcare team to have access to this data if/when they choose to discuss it with their healthcare team. The patient may only wish to share this data to be more engaged in their own health or disease management.
Suggested PGHD	A primary care provider is managing an otherwise healthy patient with chronic essential hypertension. The patient’s office-based blood pressure readings have recently been borderline high. The provider is not certain that a change in anti-hypertensive medication is warranted. The healthcare team suggests that the patient use a blood pressure cuff to record their blood pressure readings at home and share the data with their clinician during his next scheduled visit. The provider and patient discuss how the patient can monitor his blood pressure each day and identify readings that warrant a telephone call or secure message to the healthcare team. The provider confirms that the patient understands the data will not be actively monitored but will be reviewed at the next clinical visit. A summary of the agreement is documented in a clinical progress note in the patient’s electronic health record.
Solicited PGHD	A patient with congestive heart failure is hospitalized for an exacerbation of their condition. Upon discharge, the clinician enrolls the patient in a monitored remote patient monitoring program. A program care coordinator assigns appropriate FDA-cleared/approved technologies based on the patient’s specific needs and a disease management protocol (DMP). The DMP sessions collect information about symptoms, provide education, and collect biometric data (e.g., weight via a Bluetooth scale). The patient completes the DMP sessions daily, including the transmission of appropriately assigned biometric data. Session transmissions are reviewed by the care coordinator who monitors for out-of-range data and coordinates any needed changes in care with the provider and patient. The local program is responsible for this solicited PGHD program and may have developed supplemental guidelines (e.g., local staff roles and responsibilities, data review and monitoring processes, emergency protocols) in accordance with established national program guidance.

In addition to these categories, the proposed guidelines provide a framework for characterizing PGHD in the context of the level of care being provided to a patient (e.g., self-management versus case management of high-risk patients). Taken together, these considerations have important implications for patient’s expectations and the responsibility of healthcare team members to review, document, and act on shared PGHD.

## PRIORITIZING PGHD RESEARCH

PGHD can support timely data-driven decisions, such as detecting changes in a patient’s health that require intervention or treatment modification in between clinical encounters.^27^ Yet translating promising advances in technology and data tracking into successful clinical implementation requires understanding the value that PGHD can add to clinical care. While the role of PGHD in patient self-management continues to grow organically, we believe that a promising inflection point for use of PGHD lies in developing a deeper understanding of how data collection and sharing translate into actionable information that supports shared decision-making and informs clinical care in real-world settings. There are significant gaps in current knowledge about how PGHD can best be employed in the service of high-quality, patient-centered care,^28^ including a lack of evidence on effective use of PGHD and a lack of clarity about where PGHD offers the greatest impact on healthcare delivery. While many PGHD barriers have been described,^1, 29, 30^ uncertainty around the clinical value of PGHD remains a primary inhibitor for both providers and patients.^31^

Existing evidence reviews provide only limited insights on the impacts of PGHD on outcomes of care and there is a recognized need for an increased focus on measuring health-related outcomes associated with the use of PGHD. A review of the evidence for automated-entry PGHD devices and mobile apps for the prevention or treatment of 11 chronic conditions found that while randomized control trials have examined the impact of some PGHD technologies, health outcomes were generally underreported.^32^ The evidence suggested a possible positive effect of PGHD interventions on health outcomes for three chronic conditions (coronary artery disease, heart failure, asthma) and for blood pressure as a surrogate outcome for hypertension, and consistent evidence of a beneficial effect of PGHD interventions on the surrogate outcome of time to arrythmia detection.^33, 34^ Evidence for other chronic conditions was classified as unclear, hampered by the lack of reporting of health outcomes and insufficient statistical power. Until this evidence base matures, the challenge of demonstrating the clinical value of PGHD remains. A focused research agenda is crucial at this point in the evolution of PGHD to inform and expand the development of this evidence.

We propose a research agenda that reflects what we see as PGHD’s greatest potential clinical value — to support shared decision-making and inform clinical care. Realizing this value will require studies which focus on strategies to engage clinicians to (1) partner with patients to expand patient-provider communication, (2) enhance healthcare delivery, and (3) improve patient and population health outcomes. Additional research is needed to:

- Engage with clinicians to prioritize PGHD use cases and data types that are most relevant, useful, and valuable to improving patient care
- Develop and test standardized practice protocols for the optimal use of PGHD while maintaining patient safety
- Employ user-centered design to inform optimal PGHD solutions, including data capture and display, based on both patient’s and provider’s goals for data collection and use
- Examine the barriers to interoperability of PGHD with the EHR and other healthcare data sources, and develop solutions to overcome these barriers
- Explore the most effective ways to integrate PGHD into clinical workflows and decision-making processes
- Identify optimal tools and algorithms to analyze data efficiently (e.g., data visualizations) including integration with other relevant data, to improve risk prediction and diagnostic capabilities
- Evaluate the impact of PGHD on patient outcomes and effectiveness of care, including health status, quality of life, and healthcare utilization
- Study the PGHD digital divide (inequity in access to devices and access) and develop solutions to address digital disparities
- Study the implementation and scalability of successful PGHD initiatives and develop strategies to support widespread adoption and sustained use, including remuneration such as workload credit/reimbursement for review of PGHD

We note that these recommendations are synergistic with one of the top five recommended research priorities resulting from the Veterans Health Administration (VHA) State of the Art (SOTA) Conference on virtual care access, engagement, and outcomes, specifically the need for additional research to explore the integration of PGHD with EHR data to create clinically valuable predictions and alerts. VHA has great potential to study whether the sharing of PGHD can have long-term health impacts, as well as what patient populations and conditions may benefit most from its use in practice. VHA can also contribute by broadly sharing the operational guidance that has been developed, along with lessons learned in early implementation efforts.

## CONCLUSION

The ability to incorporate PGHD seamlessly into care remains limited not only due to the lack of standards for integrating disparate forms of data, but also because of the lack of evidence on effective clinical use of PGHD, and the lack of clarity about where PGHD offers the greatest impact in healthcare delivery. While recognizing the many promising aspects of PGHD, we propose an agenda for PGHD-related research in the Veterans Health Administration that emphasizes clinical value to focus attention on what we believe will be a promising inflection point for use of PGHD. More work is needed to understand how PGHD supports clinical decision-making, which, in turn, will foster both clinical adoption and patient engagement with PGHD. VHA researchers and clinicians can contribute significantly to this important need.

## References

[1] Department of Health and Human Services Office of National Coordinator for Health Information Technology (ONC). Conceptualizing a Data Infrastructure for the Capture, Use, and Sharing of Patient-Generated Health Data in Care Delivery and Research through 2024: White Paper. 2018. Available at: https://www.healthit.gov/sites/default/files/onc_pghd_final_white_paper.pdf. Accessed April 6, 2023.

[2] Austin E, Lee JR, Amtmann D (2019). Use of patient-generated health data across healthcare settings: implications for health systems. JAMIA Open..

[3] Brennan PF, Downs S, Casper G (2010). Project HealthDesign: rethinking the power and potential of personal health records. J Biomed Inform..

[4] Cohen DJ, Keller SR, Hayes GR, Dorr DA, Ash JS, Sittig DF (2016). Integrating patient-generated health data into clinical care settings or clinical decision-making: lessons learned from Project HealthDesign. JMIR Human Factors..

[5] **Casper GR, Brennan PF.** Project HealthDesign: a preliminary program-level report. AMIA Annu Symp Proc. 2013;192-199 PMC3900172 24551331

[6] **Lavallee DC, Lee JR, Austin E, Bloch R, Lawrence SO, McCall D, Munson SA, Nery-Hurwit MB, Amtmann D.** mHealth and patient generated health data: stakeholder perspectives on opportunities and barriers for transforming healthcare. mHealth. 2020;6:8. 10.21037/mhealth.2019.09.17 10.21037/mhealth.2019.09.17 PMC7063266 32190619

[7] Frühauf J, Schwantzer G, Ambros-Rudolph CM, Weger W, Ahlgrimm-Siess V (2012). Pilot study on the acceptance of mobile teledermatology for the home monitoring of high-need patients with psoriasis. Australas J Dermatol..

[8] McClellan S, Panattoni L, Chan A, Tai-Seale M (2016). Patient-initiated electronic messages and quality of care for patients with diabetes and hypertension in a large fee-for-service medical group: results from a natural experiment. Med Care..

[9] Tang PC, Ash JS, Bates DW, Overhage JM, Sands DZ (2006). Personal health records: definitions, benefits, and strategies for overcoming barriers to adoption. J Am Med Inform Assoc..

[10] Woods SS, Evans NC, Frisbee KL (2016). Integrating patient voices into health information for self-care and patient-clinician partnerships: Veterans Affairs design recommendations for patient-generated data applications. J Am Med Inform Assoc..

[11] Tan L, Hu W, Brooker R (2014). Patient-initiated camera phone images in general practice: a qualitative study of illustrated narratives. Br J Gen Pract..

[12] Drwal KR, Wakefield BJ, Forman DE, Wu WC, Haraldsson B, El Accaoui RN (2021). Home-based cardiac rehabilitation: experience from the Veterans Affairs. J Cardiopulm Rehabil Prev..

[13] Burns K, McBride CA, Patel B, FitzGerald G, Mathews S, Drennan J (2019). Creating consumer-generated health data: interviews and a pilot trial exploring how and why patients engage. J Med Internet Res..

[14] Scott D. Patient Engagement in 2019: Can it Impact Patient Outcomes? Spok. 2019, July 11. Available at: https://www.spok.com/blog/patient-engagement-in-2019-can-it-impact-patient-outcomes/. Accessed April 6, 2023.

[15] Hibbard JH, Greene J, Overton V (2013). Patients with lower activation associated with higher costs; delivery systems should know their patients’ ‘scores’. Health Aff (Millwood)..

[16] Laverty L, Gandrup J, Sharp CA (2022). Using patient-generated health data in clinical practice: How timing influences its function in rheumatology outpatient consultations. Patient Educ Couns..

[17] Bhavnani SP, Parakh K, Atreja A (2017). 2017 Roadmap for Innovation-ACC Health Policy Statement on Healthcare Transformation in the Era of Digital Health, Big Data, and Precision Health: A Report of the American College of Cardiology Task Force on Health Policy Statements and Systems of Care. J Am Coll Cardiol..

[18] Veterans Health Administration. About VA. Available at: https://www.va.gov/health/. Accessed April 6, 2023.

[19] My Health**e**Vet. Available at: https://www.myhealth.va.gov . Accessed July 20, 2023.

[20] VA Share My Health Data. Available at: https://mobile.va.gov/app/share-my-health-data. Accessed July 20, 2023.

[21] VA Annie App. Available at: https://mobile.va.gov/app/annie-app-veterans. Accessed July 20, 2023.

[22] Darkins A, Ryan P, Kobb R (2008). Care Coordination/Home Telehealth: the systematic implementation of health informatics, home telehealth, and disease management to support the care of veteran patients with chronic conditions. Telemed J E Health..

[23] Buck C, Kobb RF, Sandreth R, Alexander L, Olliff S, Westfall C, Anderson CL, Graaff AL, Giovannucci J, Rollins A (2021). Maximizing VA remote patient monitoring during the COVID-19 response. Telehealth and Medicine Today..

[24] **Armstrong CM, McGee-Vincent P, Juhasz K, Owen J, Avery T, Jaworski B, Jamison AL, Cone W, Gould C, Ramsey K, Mackintosh MA, Hilty DM.** VA Mobile Health Practice Guide (1st ed.). U.S. Department of Veterans Affairs, Washington, DC; 2021. Available at: https://connectedcare.va.gov/sites/default/files/2021-10/va-mobile-health-practice-guide.pdf . Accessed July 20, 2023.

[25] Rothenberg GM, Page J, Stuck R, Spencer C, Kaplan L, Gordon I (2020). Remote Temperature Monitoring of the Diabetic Foot: From Research to Practice. Fed Pract..

[26] Hilty DM, Armstrong CM, Edwards-Stewart A, Gentry MT, Luxton DD, Krupinski EA (2021). Sensor, wearable, and remote patient monitoring competencies for clinical care and training: scoping review. J Technol Behav Sci..

[27] **Helwig A.** Integrating PGHD into EHR: A summary of the AHRQ Project. Health Advance. 2022. Available at: https://healthcare.rti.org/insights/integrating-pghd-into-ehr-report-part-1. Accessed April 6, 2023.

[28] **Lavallee DC, Lee JR, Austin E, et al.** mHealth and patient generated health data: stakeholder perspectives on opportunities and barriers for transforming healthcare. Mhealth. 2020;6:8. 10.21037/mhealth.2019.09.17 10.21037/mhealth.2019.09.17 PMC7063266 32190619

[29] **Shaw RJ, Boazak M, Tiase V, et al.** Integrating patient-generated digital health data into electronic health records (EHRs) in ambulatory care settings: EHR vendor survey and interviews. AMIA Annu Symp Proc. 2022;439-445 PMC9285170 35854713

[30] Chung AE, Basch EM (2015). Potential and challenges of patient-generated health data for high-quality cancer care. J Oncol Pract..

[31] Adler-Milstein J, Nong P (2019). Early experiences with patient generated health data: health system and patient perspectives. J Am Med Inform Assoc..

[32] **Treadwell JR, Reston JT, Rouse B, Fontanarosa J, Patel N, Mull NK.** Automated-Entry Patient-Generated Health Data for Chronic Conditions: The Evidence on Health Outcomes. Rockville, MD: Agency for Healthcare Research and Quality (US); March 2021. Available at: https://effectivehealthcare.ahrq.gov/products/health-data-mapping/report. Accessed April 6, 2023. 33724747

[33] Reed MJ, Grubb NR, Lang CC (2019). Multi-centre Randomised Controlled Trial of a Smartphone-based Event Recorder Alongside Standard Care Versus Standard Care for Patients Presenting to the Emergency Department with Palpitations and Pre-syncope: The IPED (Investigation of Palpitations in the ED) study. EClinicalMedicine..

[34] Halcox JPJ, Wareham K, Cardew A (2017). Assessment of Remote Heart Rhythm Sampling Using the AliveCor Heart Monitor to Screen for Atrial Fibrillation: The REHEARSE-AF Study. Circulation..

